# Feasibility of mapping cross-country population coronavirus disease 2019 metrics in a federated design: learnings from a HealthData@EU Pilot use case

**DOI:** 10.1093/eurpub/ckaf017

**Published:** 2025-09-10

**Authors:** Charles-Andrew Vande Catsyne, Matilde Slot, Tamara Buble, Katrine Eriksen, Marija Svajda, Emanuel Bradasevic, Jakov Vukovic, Simon Kok Jensen, Helena Ivanković, Persephone Doupi, Christian Fynbo Christiansen, Nienke Schutte

**Affiliations:** Data Governance, Sciensano, Brussels, Belgium; CONNECT, Department of Clinical Epidemiology, Aarhus University Hospital, Aarhus, Denmark; Department of Clinical Medicine, Aarhus University, Aarhus, Denmark; Department of Public Health, Aarhus University, Aarhus, Denmark; Division for Health Informatics and Biostatistics, Croatian Institute of Public Health, Zagreb, Croatia; CONNECT, Department of Clinical Epidemiology, Aarhus University Hospital, Aarhus, Denmark; Department of Clinical Medicine, Aarhus University, Aarhus, Denmark; Division for Health Informatics and Biostatistics, Croatian Institute of Public Health, Zagreb, Croatia; Division for Health Informatics and Biostatistics, Croatian Institute of Public Health, Zagreb, Croatia; Division for Health Informatics and Biostatistics, Croatian Institute of Public Health, Zagreb, Croatia; CONNECT, Department of Clinical Epidemiology, Aarhus University Hospital, Aarhus, Denmark; Department of Clinical Medicine, Aarhus University, Aarhus, Denmark; Division for Health Informatics and Biostatistics, Croatian Institute of Public Health, Zagreb, Croatia; Data and Analytics Department, Finnish Institute for Health and Welfare, Helsinki, Finland; CONNECT, Department of Clinical Epidemiology, Aarhus University Hospital, Aarhus, Denmark; Department of Clinical Medicine, Aarhus University, Aarhus, Denmark; Data Governance, Sciensano, Brussels, Belgium

## Abstract

The European Health Data Space aims to transform health data management across the EU, supporting both primary and secondary uses of health data while ensuring trust through General Data Protection Regulation compliance. As part of the HealthData@EU Pilot, this study investigates coronavirus disease 2019 (COVID-19) testing, vaccination, and hospitalization metrics across six European countries, with a focus on socioeconomic disparities and challenges in cross-border data access and standardization. This observational, retrospective cohort study used a federated analysis framework across Belgium, Croatia, Denmark, Finland, and France. Data were linked from administrative, social, health, and care records within each country’s trusted research environment. A Common Data Model (CDM)-guided data harmonization, enabling nodes to perform independent analyses and share aggregated results. Key data processes (discovery, access, preparation, and analysis) were decentralized, with significant variability in data access procedures, security protocols, and available resources among nodes. The study revealed substantial differences in COVID-19 testing, vaccination, and hospitalization rates across countries. Denmark exhibited notably higher testing and infection rates. However, the study encountered key challenges: complex data access procedures, fragmented and incomplete socioeconomic data, and the need for extensive harmonization. Learnings from this pilot underscore the importance of streamlined, cross-country data access and standardization processes, which the European Health Data Space (EHDS) framework aims to address. The pilot demonstrates the feasibility of federated health data analysis across multiple countries while highlighting limitations in data access and interoperability. The EHDS framework offers a promising path to overcome these barriers, supporting efficient and standardized cross-border health research in the EU.

## Introduction

In spring 2024, the European Parliament and Council agreed on the Commission's proposal for the European Health Data Space (EHDS) [1]. This initiative will transform health data management across the EU, empowering individuals to control their data while enabling seamless data exchange for healthcare (primary use) and fostering a unified market for electronic health records. It also supports the reuse of health data for research, innovation, policy-making, and regulatory purposes (secondary use).

Trust is central to EHDS, which builds on key EU regulations like the General Data Protection Regulation (GDPR) [2], and the Data Governance Act [3] or Data Act [4], introducing sector-specific rules for sensitive health data. The EHDS regulation also includes opt-out options, balancing patient preferences with the need for public interest data access.

The HealthData@EU Pilot project [5], launched in October 2022, is a 2-year initiative aimed at investigating and establishing an infrastructure for the EHDS, specifically focused on the secondary use of health data. Co-financed by the EU4Health programme [6], this project brings together 17 partners, including health data access bodies, research infrastructures, and European agencies, to create a pilot version of the EHDS infrastructure and provide initial guidelines for data standards, data quality, data security, and data transfer to support this cross-border infrastructure.

To demonstrate the feasibility and potential of reusing health data across multiple European countries, the HealthData@EU Pilot is running five use cases selected by the European Commission. These use cases are designed to showcase how health data can be utilized for public health, research, and innovation. The lessons learned from these use cases will inform the development of services at both the national node and central service levels.

This paper presents one of these real-world use cases. It aims to determine population uptake metrics on coronavirus disease 2019 (COVID-19) testing, vaccination, and hospitalization stratified by socioeconomic indicators. It also aims to explore the specific challenges of linking clinical and socioeconomic data from multiple datasets within each country across multiple countries. Moreover, the paper evaluates the feasibility and effectiveness of using a federated design to conduct this research, providing valuable feedback, and insights on where in the user journey the EHDS can make an impact.

## Methods

### Study design

This study is an observational, retrospective, longitudinal (cohort) study utilizing routinely recorded administrative, social, health, and care data from multiple countries/regions in Europe. Various existing databases were linked to address the research objectives, covering sources of individual-level demographic, socioeconomic, hospital (clinical or insurance data), and laboratory test result data.

This study employed a client-server federation methodology previously implemented in the PHIRI project [7], also adopted in other large EU projects, such as BY-COVID [8], where a central node orchestrated the workflow and the communication between federated nodes (in the different countries) that have access to individual-level health patient data (sensitive health data). The workflow was adapted from Aldridge et al. [9] and González-García et al. [10].

**Figure 1.**
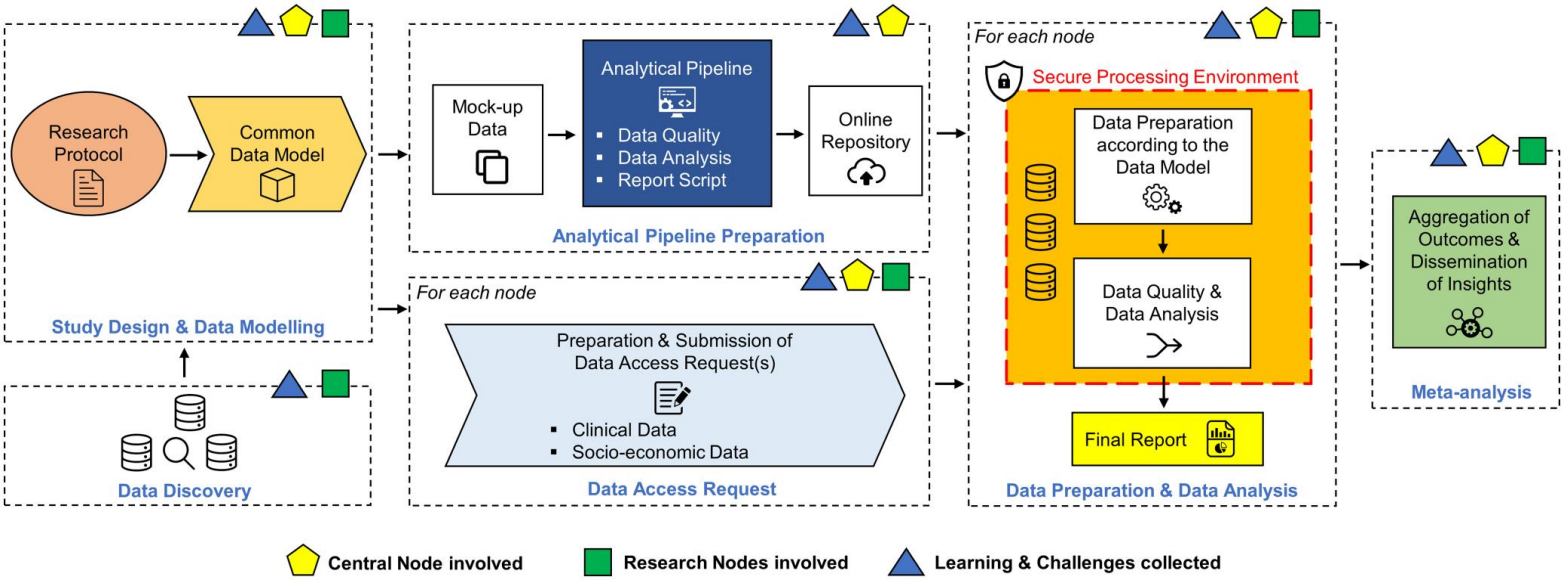
Adapted methodological stepwise workflow for federated research. (This figure is an adaptation of the Figure 1 published in ‘PHIRI: Lessons for an extensive reuse of sensitive data in federated health research’ [10].) The research process began with the formulation of eligibility criteria and research questions, defining the study cohort, outcomes, and ensuring clear data requirements. A collaborative effort between the central node and the research nodes (Nodes, Table S1) translated these requirements into a CDM. The central node, which serves as the coordinating hub, was responsible for facilitating collaboration between research nodes, defining the research protocols, translating them into the CDM, and developing the analytical pipeline. The central node also prepared mockup data and scripts for data quality checks, analysis, and reporting, which were shared via an online repository for deployment across SPE at each research node. The research nodes, which represent individual entities (e.g. institutions, organizations, or countries), retained ownership of their data and operated autonomously within their SPEs. Each research node prepared and analysed its local data according to the CDM using the shared pipeline. They then submitted aggregated results, such as summary statistics or model updates, to the central node. Following data preparation and analysis at each node, the central node aggregated the results, performed meta-analysis, and disseminated the overall insights. Throughout the process, challenges and learnings were documented.

The research process started by formulating eligibility criteria and research questions defining the cohort and outcomes, ensuring clear data requirements. A federated discussion between the research teams (Nodes, Table S1) translated this into a Common Data Model (CDM), which was used to develop an analytical pipeline, including data quality, analysis, and reporting scripts. Mockup data and scripts were shared via a central repository for fast deployment in secure processing environments (SPEs). Each node analysed its data, and the results were sent to the central node, where support and coordination were provided throughout the process. Challenges were addressed in regular coordination meetings (Fig. 1).

### Data sources

The data sources used are summarized (Table S1), providing an overview of the key databases employed in the study.

Each node participating in the study built a national cohort by linking individual-level pseudonymized health data with socio-economics records within their trusted SPE. These cohorts adhered to a shared data model to ensure consistency across all regions. This method enabled the production of aggregated data that could be used for detailed analysis or meta-analysis, all while ensuring compliance with the GDPR and local data privacy laws.

### Study population

The enrolment period started from the beginning of the SARS-CoV-2 vaccination campaign (1 January 2021) through to 31 December 2022.

The countries enrolled in this study were Croatia, Belgium, France, Denmark, and Finland.

The source population for the study was the entire population, including both men and women aged 18–115 years old who were living in the respective countries at the start of the enrolment period. To construct the cohort, we included only individuals who had been tested at least once for COVID-19 using a registered polymerase chain reaction (PCR) test and/or had received at least one dose of a COVID-19 vaccine. Individuals who died during the study period, as well as those who neither received a registered vaccine dose nor underwent a PCR test during the study period, were excluded. While the analysis was conducted on this filtered cohort, the full population data were necessary to apply these inclusion criteria accurately.

### Variables

We assessed our outcome variables using metrics for testing, vaccination, and hospitalization. Testing metrics were represented by the median number of total PCR tests conducted within each study population, along with the median number of positive PCR tests among those tested.

Vaccination metrics reflected the COVID-19 vaccination rate within each study population. A COVID-19 vaccination is defined as receiving a complete vaccination scheme (either two doses plus a booster for BioNTech-Pfizer, Moderna, AstraZeneca, or Novavax vaccines, or one dose plus a booster for the Johnson & Johnson vaccine).

Hospitalization metrics captured the percentage of COVID-19-related hospitalizations within each study population. Due to the lack of consensus among the nodes regarding the definition of COVID-19 hospitalization in their respective databases, a COVID-19 hospitalization was defined as any hospitalization occurring within 14 days of a positive PCR test.

Various sociodemographic and socioeconomic factors captured at the start of the study period [sex, age (e.g. 18–25, 25–35…)], residence area (NUTS2), education level [11], income category [12], migration background [13], and household type [14]) were investigated in stratified analyses. More information available in Table S2.

The exposures were the testing percentage, the presence of COVID-19 infection determined by a positive PCR test and the COVID-19 vaccination status in both study population and vaccinated population.

### Statistical methods

The statistical analysis used available case analysis (pairwise deletion), excluding individuals only if they had missing data for the specific variable being analysed. Normality of age distribution within each cohort was assessed by Q–Q plot. Total individuals per group, medians [interquartile range (IQR)] and percentages were generated within each use case partner’s SPE and only aggregated results were exported. The data analysis was performed using R (v4.2.0) [15] and RStudio (2024.04.0) [16], and the final report was generated as a Quarto interactive HTML document.

While comparisons were made across nodes, the objective was not to imply that one country outperforms another. Differences in healthcare infrastructure, policies, and socioeconomic contexts among countries inherently influence access to testing, vaccination, hospitalization, and other variables. Our aim is solely to observe and report the results, focusing on descriptive analysis rather than drawing causal inferences or conducting tests to determine correlations between factors in this observational study.

### Privacy and security measures

To protect individual-level data and to prevent re-identification, a federated approach was used (Fig. 1), ensuring sensitive data remained under the control of the original data holders, with only analysis code and results shared. Access to data was limited to the necessary variables required for the CDM, following the principle of data minimization. Total number of individuals was rounded to the nearest hundred, and a minimum threshold of 10 individuals per group was enforced. Groups or subgroups with fewer than 10 individuals were excluded to prevent exposure to small group statistics, ensuring robust privacy protection.

### Ethics and permissions

Each node acquired the necessary approvals to meet their relevant Ethic Committee requirements if it was necessary. All the general data protection regulations (GDPR) are the responsibility of the node (national nodes, research team, and/or data holders). Individual written patient consent was not required for this study.

## Results

### Challenges in data access and completeness

Of the five nodes, only three have managed to access the requested data at the time of this writing (Table S3). The Danish and Belgium nodes were the only ones so far able to perform the analysis on all clinical data and socioeconomic data required for the study analysis.

The Danish node did not encounter any problems with data application/access, probably because Denmark has established nationwide registries on SPE and because the researchers are experienced in registry-based studies.

For the Belgium node, the research team initially obtained access to the entire study population. However, authorization to process data from the Flanders region was delayed by 11 months without explanation, despite simultaneous data access requests for all regions.

The Croatian node encountered challenges in accessing socioeconomic data, which resulted in the provision of only clinical data. While migration background is unavailable in the required format, the research team contacted the Tax Administration and Statistics Bureau for the other metrics (household type, income, and education level). Both declined to provide individual-level data due to legal restrictions. The Tax Administration cited confidentiality under the General Tax Law, while the Statistics Bureau referenced the classified nature of census data. The Statistics Bureau suggested including the variables in the Annual Implementation Plan, this process would extend beyond the project's timeline. Finally, it was necessary to request and obtain approval from the Croatian Institute of Public Health (CIPH) Ethics Committee for the research, which took 6 months.

At the time of authoring, the Finnish node was working to secure access to necessary data, with socioeconomic variables posing the main challenge. The team decided to first apply for an internal research permit for health-related variables available in Finnish Institute for Health and Welfare (THL) registries, planning a subsequent application to Statistics Finland for socioeconomic data upon positive feedback. Alternative options, like using ready-made datasets from Findata [17] that combine THL data with other sources, were also considered. However, suitable datasets were not yet available: For instance, the COVID-19 dataset did not fit the study timeframe, and FinRegistry datasets [18] were still unavailable and unlikely to meet the use case needs.

The French node was rejected during the data application process for several reasons: First, the lack of individual-level socioeconomic data, necessitating analysis based on local-level aggregated data, introduced significant bias and reduced result relevance. Additionally, despite extensive adaptation of study protocol, the difficulty of mobilizing national expertise meant the submitted protocol lacked the required quality and detail; the ethical committee highlighted methodological issues, unclear sections, and insufficient justification. The large number of patients involved (12 million, the maximum ever approved by Health Data Hub) made the committee reluctant to grant a favourable opinion without a complete methodological overhaul. Given extensive feedback and fundamental methodological concerns, the team deemed further data authorization pursuit unreasonable.

### Data standardization issues across countries

Several challenges were identified by the nodes during the data standardization process, which introduced potential biases into the subsequent analysis.

The Belgian node relied on the national registry to provide demographic variables that are updated monthly. As a result, COVID-19 clinical data from the study period (2021–22) had to be linked with national registry data from the data analysis period (2024). Furthermore, the income category variable represented the entire household rather than individual members.

Similarly, the Croatian node relied on the population database available to CIPH to provide information on demographic variables and residential status. The data in this database are updated daily, and there is no historical tracking of address changes. Consequently, clinical data from the study period (2021–22) was linked with data from 2024, which was available at the beginning of the data preparation process. While previous findings suggest that the residential status of individuals in the database infrequently changes, this may not accurately reflect individuals' actual status.

While the definition of COVID-19 hospitalization remained consistent across all nodes, the criteria for hospitalization itself varied. In Croatia, data targeted inpatient admissions only. In Belgium and Denmark, hospitalization was defined as stays of at least 24 hours.

The SPE specifications were not standardized across nodes, and certain nodes, such as the Belgian SPE, supported a more limited range of data analysis tools, with R being the only practical option. As a result, the entire analytical pipeline had to be developed in R to accommodate these constraints.

### Mapping process, time, and resources

The central node finalized the research protocol within 1 month, followed by another month dedicated to implementing the CDM. The development of the analytical pipeline required the most time, taking 3 months to complete, ensuring that all nodes could align with the agreed-upon methods and standards for data analysis.

We compiled the time required for each step of the use case journey, including data discovery, data access procedures, data preparation, and data analysis, across the different nodes. This allows for a clearer understanding of the distribution of time and resources involved in managing the data for this study (Fig. S1).

It is important to note the mentioned steps were global processes that did not involve the research teams working full-time on these tasks but were instead conducted alongside other responsibilities.

While no fee was applied for accessing data in Croatia, the other two nodes that accessed data encountered significant access fees.

### Descriptive data

**Figure 2.**
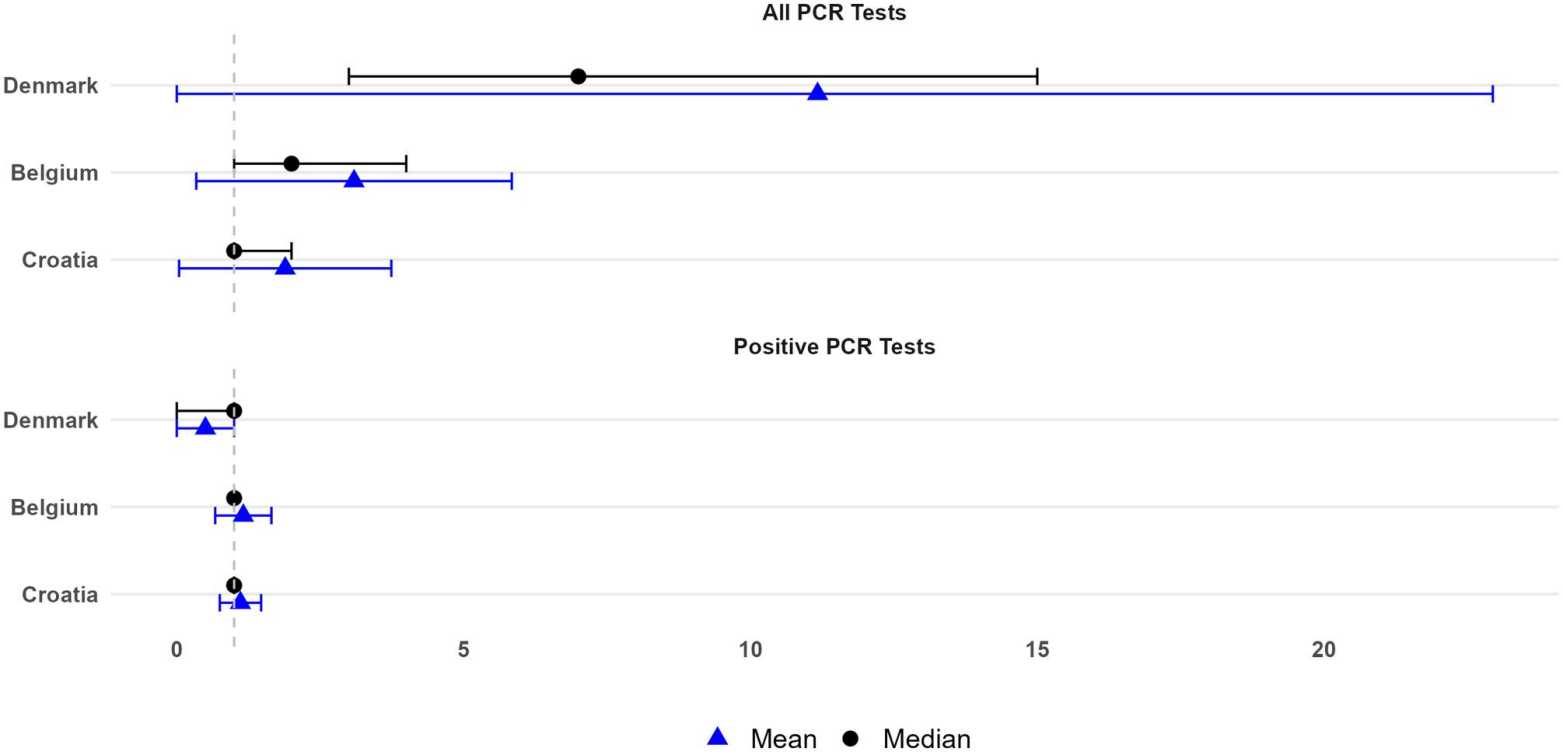
COVID-19 PCR testing metrics across countries. The mean (▲) and median (●) values for the total number of PCR tests (top panel) and positive PCR tests (bottom panel) are shown for Denmark, Belgium, and Croatia. The denominator for all PCR tests includes all individuals who had at least one PCR test performed. For positive PCR tests, the denominator consists of all individuals who received at least one positive PCR test result. Horizontal lines represent the interquartile range for median values (●) and standard deviation (SD) for mean values (▲). The vertical dashed line at one is used as a reference point.

**Figure 3.**
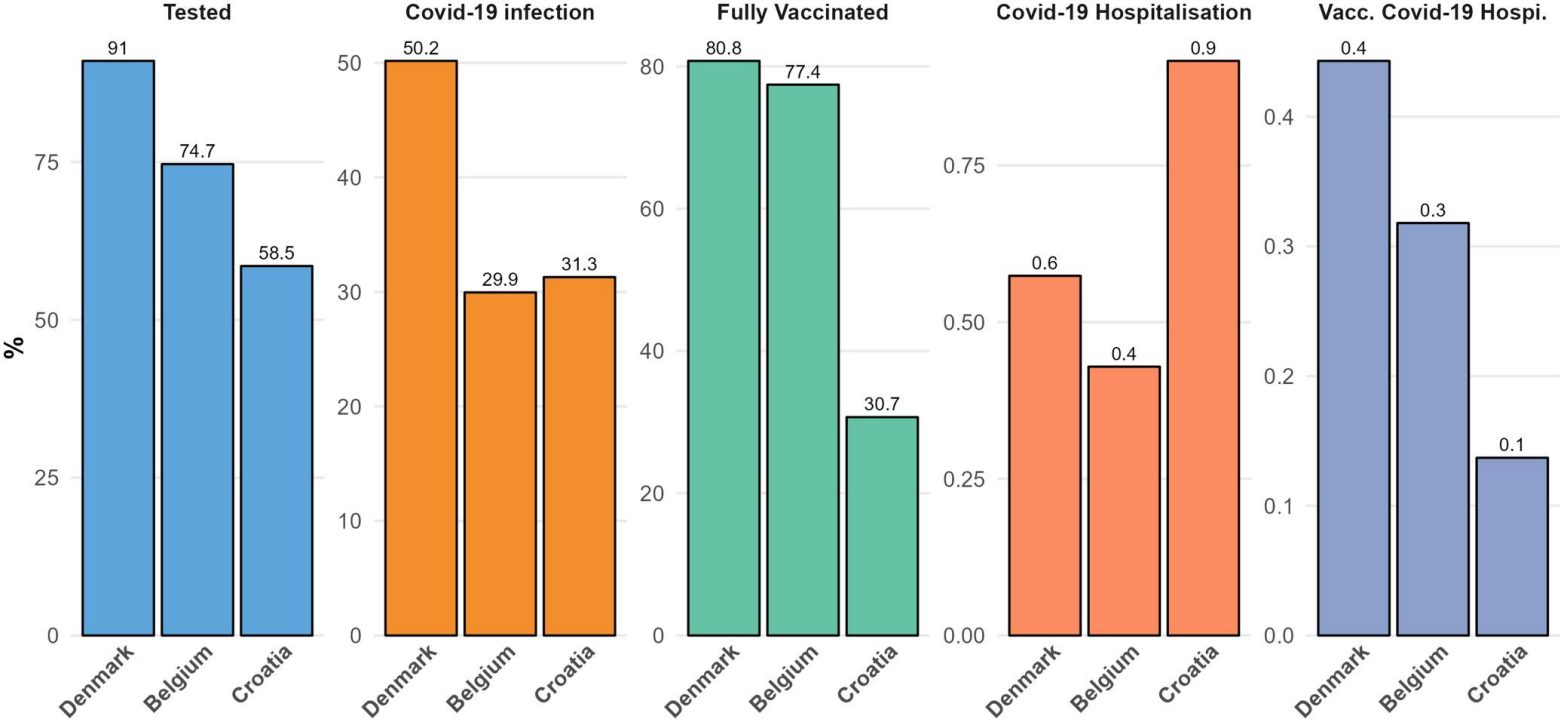
COVID-19 testing, vaccination, and hospitalization metrics by country. This figure displays the percentage of the population in Denmark, Belgium, and Croatia that underwent PCR testing, tested positive for COVID-19, were fully vaccinated, were hospitalised due to COVID-19, and were hospitalised due to COVID-19 after vaccination. From left to right, the panels represent the percentage of individuals tested via PCR, the percentage with a positive PCR result, the percentage fully vaccinated, the percentage hospitalized due to COVID-19, and the percentage of fully vaccinated individuals hospitalized due to COVID-19.

Across the study period, the study population sizes were 4 480 900 in Denmark, 8 791 300 in Belgium, and 2 774 900 in Croatia. Denmark had the highest mean number of PCR tests per individual (11.2), compared to Belgium (3.1) and Croatia (1.9) (Fig. 2). Proportion with COVID-19 infection was also highest in Denmark (50.2%), followed by Croatia (31.9%) and Belgium (30.0%). Vaccination uptake was high in Denmark and Belgium, with 80.8% and 77.4% fully vaccinated, respectively, while Croatia’s vaccination uptake was lower at 30.69%. Proportion of COVID-19 hospitalizations in the overall cohort was the highest in Croatia (0.9%), while hospitalizations in vaccinated cohort was the highest in Denmark (0.4%) (Fig. 3).

Testing metrics by age group showed lower average and median PCR testing among individuals aged 65–95 in Denmark. In contrast, older age groups in Belgium and Croatia were tested more frequently, proportionally, than younger individuals (Fig. S2). Individuals aged 55–95 had the highest vaccination uptake and COVID-19 hospitalization percentages across all countries (Fig. S3).

**Figure 4.**
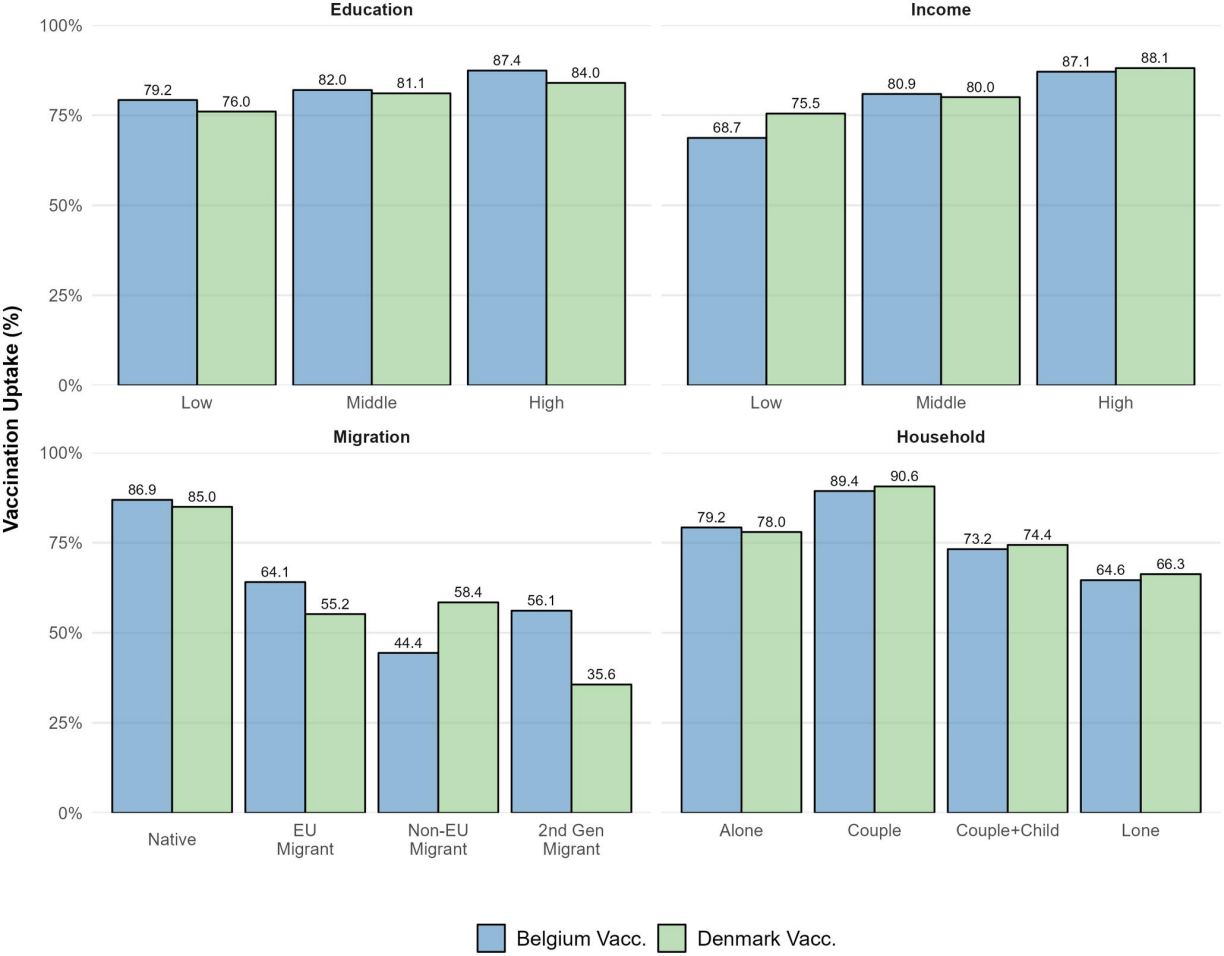
Proportion of COVID-19 vaccination uptake by socioeconomic indicators in Denmark and Belgium. The bar charts represent the percentage of vaccination uptake among various socioeconomic groups, categorised by education level, income level, migration background, and household type. Vaccination uptake percentages are shown on the left *y*-axis. Croatian data are not included due to missing socioeconomic data.

Proportion of vaccination uptake and COVID-19 hospitalization between Belgium and Denmark, segmented by education, income, migration background, and household type is available in (Fig. 4 and Fig. S4).

All metrics are provided as Supplementary Material (Tables S4–S6).

## Discussion

This study was conducted within the context of the HealthData@EU Pilot project, which provided valuable insights into cross-border data standardisation and secure sharing.

We observed differences in testing policies, with Denmark’s high testing levels likely contributing to a higher observed proportion of COVID-19 infections [19, 20]. Vaccination was associated with reduced COVID-19 hospitalization percentages across all countries, aligning with prior evidence supporting the protective effect of vaccination against severe outcomes [21, 22]. COVID-19 hospitalizations were nearly twice as high in Croatia, possibly due to lower vaccination uptake during the study period. Disparities in vaccination rates were evident, with disadvantaged groups (lower income, lower education, and immigrant backgrounds) less likely to be vaccinated [23]. These findings underscore the influence of socioeconomic status on vaccine access and uptake, though excluding deceased individuals may bias results toward healthier cohorts. Further in-depth analyses and statistical tests are needed to identify specific correlations and better understand the underlying factors driving these disparities. The difference-in-differences method, for example, may help clarify the role of socioeconomic factors and systemic differences, offering a better understanding of the relationships underlying these disparities across diverse healthcare systems.

A federated approach brings several advantages, particularly in terms of data privacy and security. By keeping sensitive individual-level data decentralized within trusted environments, the approach minimizes risks of data breaches and ensures compliance with privacy laws and data protection regulations (‘data visiting principle’). Additionally, the federated approach is cost-effective and time-saving, as shared tools and workflows enable quicker deployment and allow each node to process data independently, without depending on others’ progress. The system’s scalability also makes it suitable for large-scale, global research projects, as it can accommodate new nodes without significant structural changes. However, there are limitations to this approach. The federated model requires significant technical expertise and coordination, as each node must have qualified experts.

Furthermore, to ensure the analytical pipeline functions within each SPE, it must align with their varying specifications. SPEs differ widely in tools, procedures, data extraction timelines, and available resources, complicating the design of a consistent analysis process across all nodes. Providentially, the EHDS regulation will, by means of an implementing act, provide for the technical, organizational, information security, confidentiality, data protection, and interoperability requirements for the SPEs, including the technical characteristics and tools. This will support the alignment of SPE’s, making them suitable for safe processing of health data.

Several challenges emerged that impacted the ability to access and analyse data across different nodes.

One of the first challenges concerns data discovery due to the lack of comprehensive data catalogues, including clear descriptions of datasets, their available variables, and access procedures. The data discovery process can be time-consuming, as researchers often navigate incomplete metadata typically available in static files rather than through accessible and searchable online metadata catalogues. Moreover, metadata frequently lacks detailed information about key data aspects, particularly data quality. This often leads to unexpected challenges when access to the data is finally granted, as issues with variable definitions and data quality only become apparent during the data preparation phase. This underlines the importance of involving ‘local’ experts with in-depth knowledge of the data structure and quality from each country to address these challenges effectively.

Another issue faced by the nodes was the fragmentation and incompleteness of data across different countries, leading to gaps and biases that complicate cross-country comparisons. Data were often inconsistently captured or managed by different data holders, resulting in delays or obstacles in obtaining the necessary linked data sets. Socioeconomic information was sometimes only available at broader area levels, limiting its analytical value. Additionally, technical or legal restrictions often prevented the linking of datasets, or accessing certain socioeconomic data required separate application processes, except for specific statistical purposes. Navigating diverse data access procedures involving various administrative, legal, and regulatory hurdles was one of the biggest and most lengthy challenges we faced in this use case. These delays emphasize the critical need for transparent and harmonized data access procedures across countries.

Disparities in data access costs exist, with some datasets offered for free while others involve substantial costs. This lack of transparency and standardization poses a significant barrier to equitable access, particularly for researchers and institutions with limited resources. To address these challenges, a standardized regulatory framework is needed to harmonize data access policies and ensure affordability. Such a framework should cap fees, establish transparent cost structures, and promote subsidized or free access for non-commercial research focused on public health. Additionally, the development and dissemination of open-access synthetic datasets—statistically representative yet privacy-preserving—could democratize access to health information, offering a low-cost alternative for many types of analysis.

Cross-country standardization of the data was enabled by the use of a CDM, allowing for a smooth exchange of information, and ensuring correct variable selection, harmonization, and analysis across countries, leading to reliable and comparable results. However, as each data holder is currently applying different standards and coding systems, standardization through the CDM was an iterative and time-consuming process: The data preparation phase was the second longest part of the use case journey.

The challenges encountered during this study highlight several areas that the EHDS regulation aims to address to facilitate efficient and equitable cross-border health research. The EHDS regulation provides for the creation of a central metadata catalogue with unified dataset descriptions, allowing researchers to better understand available variables and data quality before accessing datasets. It also introduces a unified data application form, enabling researchers to apply for permits to process multiple datasets across different countries, thereby reducing delays and improving the efficiency of multinational studies.

Furthermore, a standardized regulatory framework is needed to harmonize data access policies and ensure affordability. The European Commission will, by means of EHDS implementing acts, lay down principles for the fee policies and fee structures. We argue that this framework should establish transparent cost structures, cap excessive fees, and promote subsidized or free access for non-commercial research focused on public health. Additionally, promoting the development and dissemination of open-access synthetic datasets (statistically representative yet privacy-preserving) could democratize access to health information and provide a low-cost alternative for many types of analyses.

While the EHDS does not directly tackle interoperability at the data level for secondary use of data, focusing instead on metadata, it lays the groundwork for further harmonization of standards. It also enables data holders to provide essential information about coding systems and variables, which will support more consistent and reliable data integration in the future.

## Supplementary Material

ckaf017_Supplementary_Data

## Data Availability

All data are incorporated into the article and its online supplementary material.

## Disclaimer

Key points

- Differences in COVID-19 testing policies complicate meaningful cross-country comparisons of health data.
- Socioeconomic disparities impact vaccine uptake, underscoring challenges in achieving equitable healthcare access.
- Fragmented and incomplete data hinder cross-country research, highlighting the need for standardized data sharing.
- European Health Data Space hold promise for addressing challenges posed by varied national policies, offering potential relief and harmonization in the future.
- Federated data models enhance privacy but require complex coordination, affecting the scalability of international health research.
