# Supplementary figures and images for: Feasibility of mapping cross-country population coronavirus disease 2019 metrics in a federated design: learnings from a HealthData@EU Pilot use case
Source: Eur J Public Health. 2025 Sep 10;35(Suppl 3):iii11–7. doi: 10.1093/eurpub/ckaf017 (PMC12420901; doi:10.1093/eurpub/ckaf017)

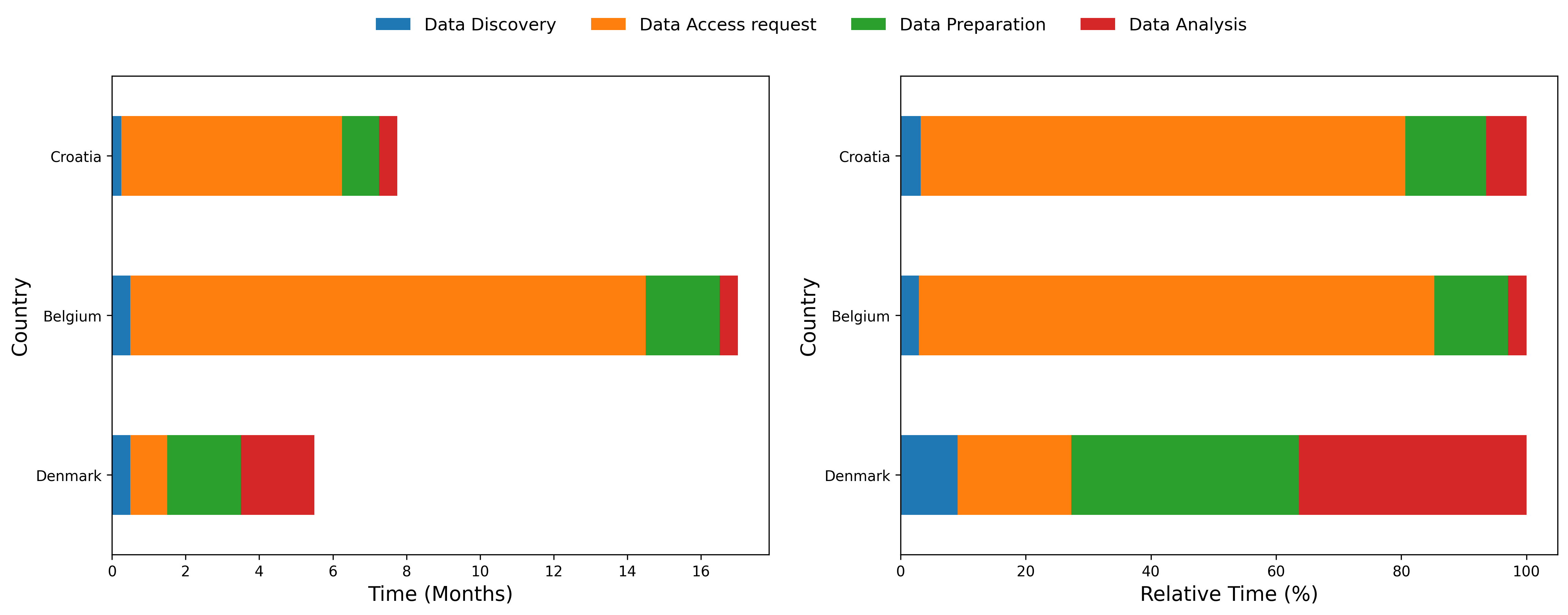

Supplement: ckaf017_Supplementary_Data [file ckaf017_supplementary_data.zip › ckaf017_Supplementary_Data/ejph-2024-11-om-0786-File006.tif]

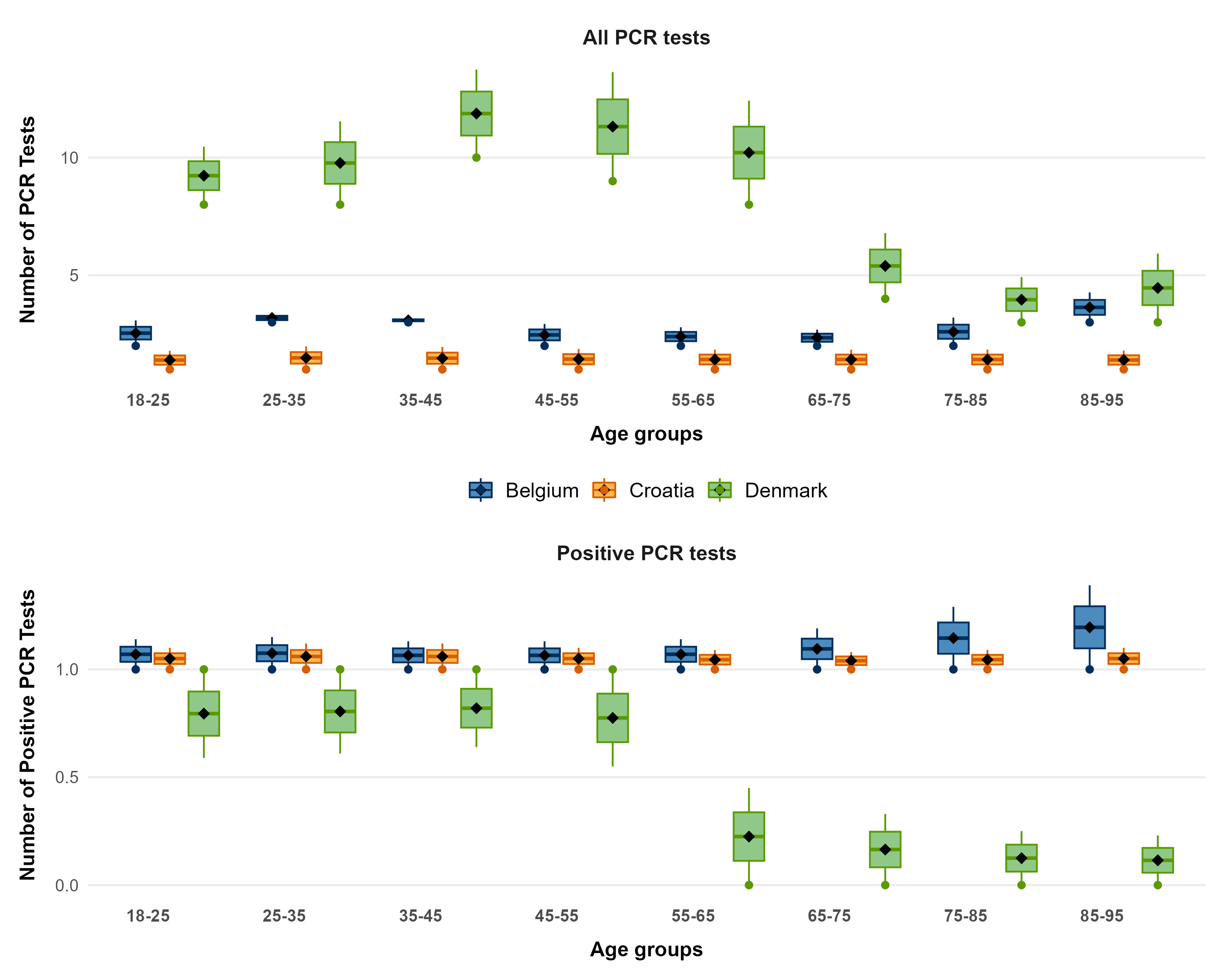

Supplement: ckaf017_Supplementary_Data [file ckaf017_supplementary_data.zip › ckaf017_Supplementary_Data/ejph-2024-11-om-0786-File007.tiff]

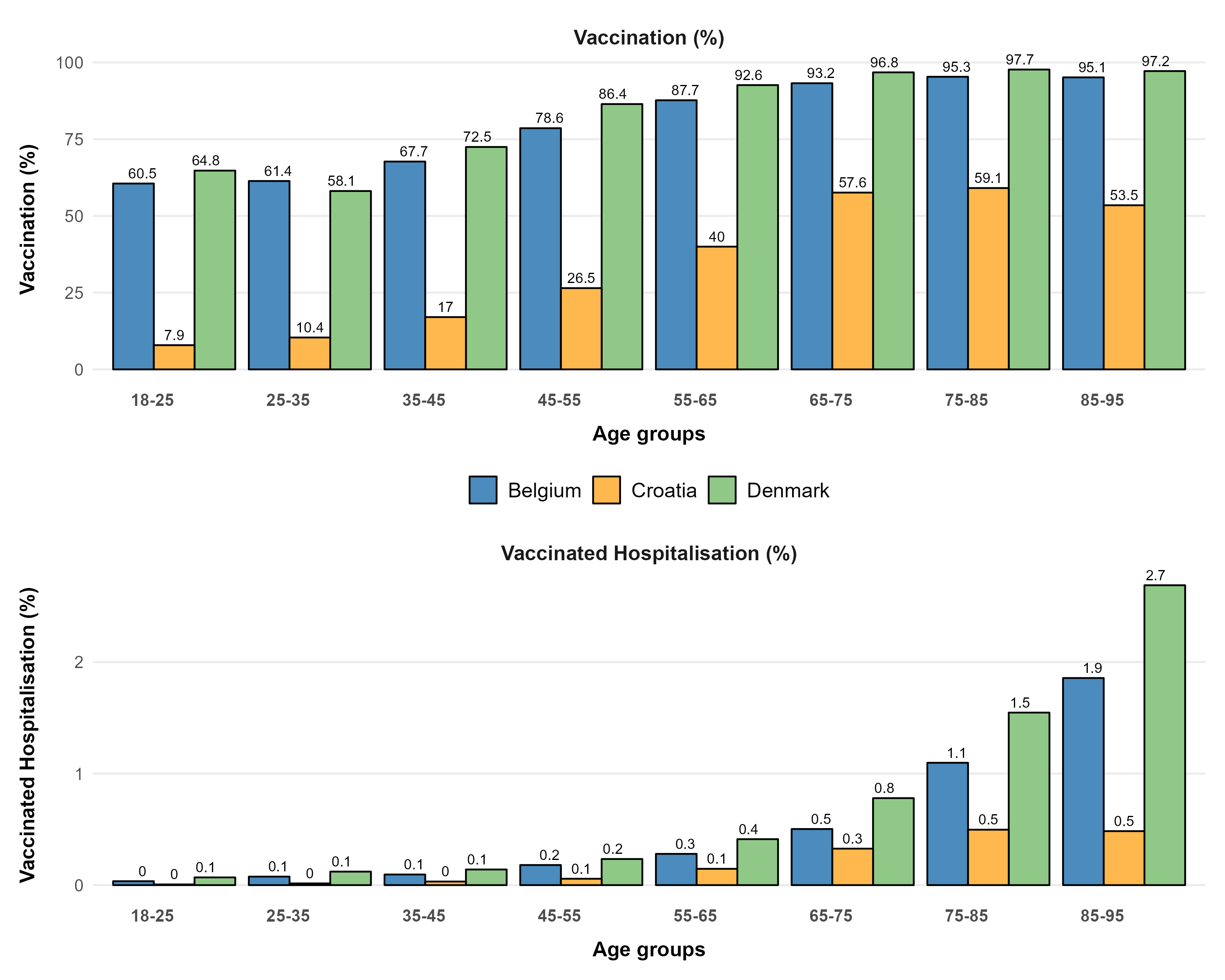

Supplement: ckaf017_Supplementary_Data [file ckaf017_supplementary_data.zip › ckaf017_Supplementary_Data/ejph-2024-11-om-0786-File008.tiff]

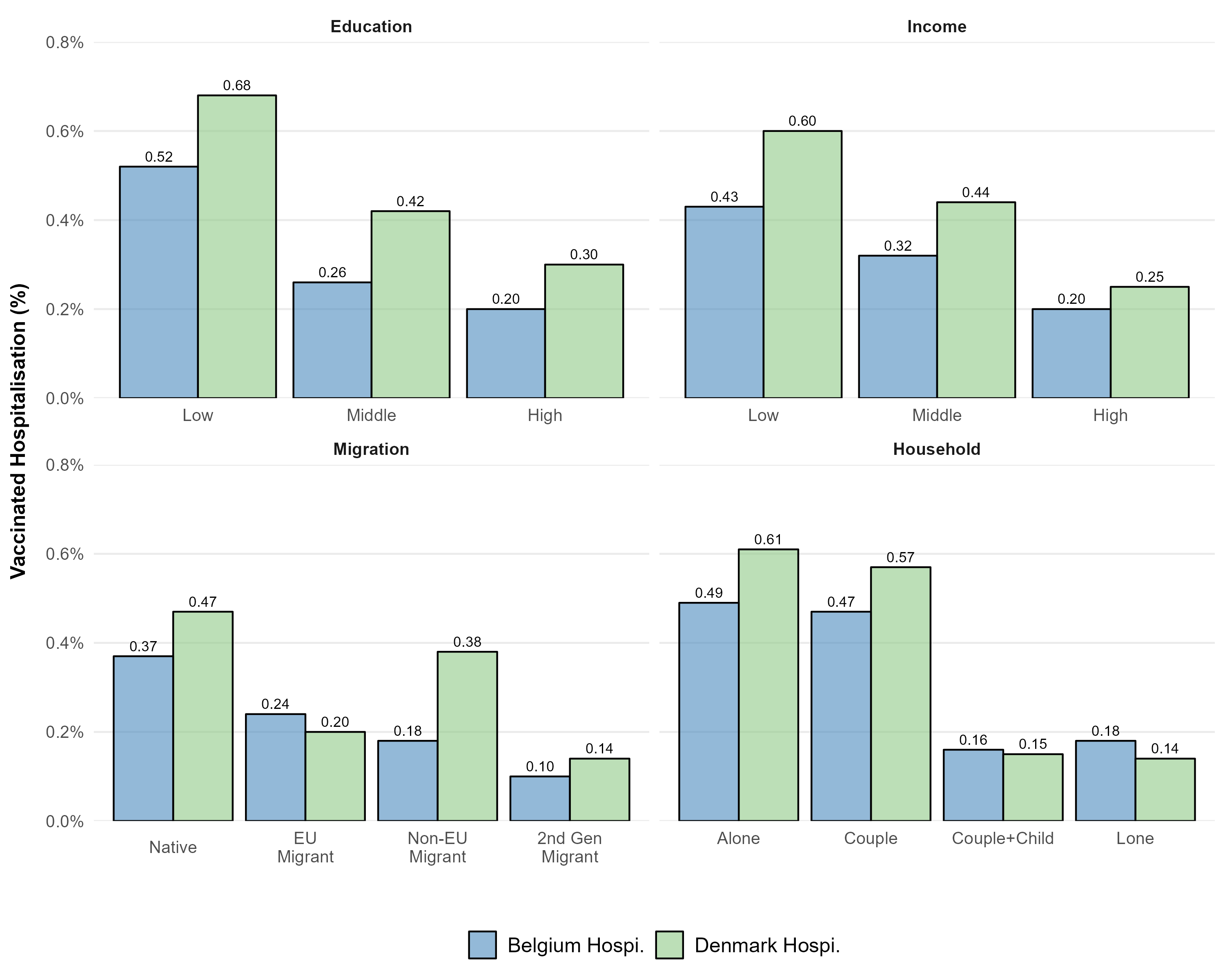

Supplement: ckaf017_Supplementary_Data [file ckaf017_supplementary_data.zip › ckaf017_Supplementary_Data/ejph-2024-11-om-0786-File009.tiff]
